# Benchmarking online food delivery applications against menu labelling laws: a cross-sectional observational analysis

**DOI:** 10.1017/S1368980024000673

**Published:** 2024-04-01

**Authors:** Sophia Cassano, Anna Jia, Alice A Gibson, Stephanie R Partridge, Virginia Chan, Penny Farrell, Philayrath Phongsavan, Margaret Allman-Farinelli, Si Si Jia

**Affiliations:** 1 The University of Sydney, Sydney Nursing School, Faculty of Medicine and Health, Sydney, NSW 2006, Australia; 2 The University of Sydney, Menzies Centre for Policy and Economics, School of Public Health, Faculty of Medicine and Health, Sydney, NSW 2006, Australia; 3 The University of Sydney, Charles Perkins Centre, Sydney, NSW 2006, Australia; 4 The University of Sydney, Engagement and Co-Design Research Hub, School of Health Sciences, Faculty of Medicine and Health, Sydney, NSW 2006, Australia; 5 The University of Sydney, Prevention Research Collaboration, Sydney School of Public Health, Faculty of Medicine and Health, Sydney, NSW 2006, Australia

**Keywords:** Food environment, Online food delivery, Menu labelling, Nutrition policy, Energy labelling, Calorie labelling

## Abstract

**Objective:**

It is unknown how well menu labelling schemes that enforce the display of kilojoule (kJ) labelling at point-of-sale have been implemented on online food delivery (OFD) services in Australia. This study aimed to examine the prevalence of kJ labelling on the online menus of large food outlets with more than twenty locations in the state or fifty locations nationally. A secondary aim was to evaluate the nutritional quality of menu items on OFD from mid-sized outlets that have fewer locations than what is specified in the current scheme.

**Design:**

Cross-sectional analysis. Prevalence of kJ labelling by large food outlets on OFD from August to September 2022 was examined. Proportion of discretionary (‘junk food’) items on menus from mid-sized outlets was assessed.

**Setting:**

Forty-three unique large food outlets on company (e.g. MyMacca’s) and third party OFD (Uber Eats, Menulog, Deliveroo) within Sydney, Australia. Ninety-two mid-sized food outlets were analysed.

**Participants:**

N/A.

**Results:**

On company OFD apps, 35 % (7/23) had complete kJ labelling for each menu item. In comparison, only 4·8 % (2/42), 5·3 % (2/38) and 3·6 % (1/28) of large outlets on Uber Eats, Menulog and Deliveroo had complete kJ labelling at all locations, respectively. Over three-quarters, 76·3 % (345/452) of menu items from mid-sized outlets were classified as discretionary.

**Conclusions:**

Kilojoule labelling was absent or incomplete on a high proportion of online menus. Mid-sized outlets have abundant discretionary choices and yet escape criteria for mandatory menu labelling laws. Our findings show the need to further monitor the implementation of nutrition policies on OFD.

In 2017, overweight and obesity affected 25 % of children and adolescents and 67 % of adults living in Australia^(1)^. Obesity is associated with poor health outcomes and higher risk of non-communicable diseases such as CVD, diabetes and some cancers^(2,3)^. The development of overweight and obesity is attributed to multiple individual and environmental factors, including the availability of energy-dense foods, abundant food supply and appealing marketing of unhealthy foods and drinks^(3)^. A key dietary risk factor for obesity and chronic diseases is the excess consumption of discretionary foods, which are energy-dense and nutrient-poor foods that are not essential for health and yet high in saturated fat, added sugars, Na or alcohol as defined by the Australian Dietary Guidelines^(4)^. In 2018, discretionary foods contributed a third of total daily energy intake for Australians^(5)^ and findings from Australia’s Household Expenditure Survey in 2015–2016 showed that meals-out and fast foods accounted for, on average, 34 % of weekly budgets for foods and beverages^(6)^. Research has shown that a high consumption of takeaway foods is associated with poorer diet quality and higher prevalence of abdominal obesity in young adults^(7)^.

The digitalisation of the food environment is changing the way individuals order and access foods, with potentially harmful implications for public health^(8)^. Online food delivery (OFD) services are websites or applications that allow consumers to order food and beverages to be picked up or delivered by couriers^(9)^. OFD connect consumers to a wide range of food outlets and can include a food outlet’s company apps (e.g. McDonald’s ‘MyMacca’s’ app) or third-party platforms such as Uber Eats^(9)^. Globally, OFD were used by over 1·8 billion people in 2022^(10)^ and are expected to grow to 2·64 billion users by 2027^(9)^. In Australia, the market-leading third-party OFD include Uber Eats, Menulog and Deliveroo accounting for 59·7 %, 17·5 % and 12·1 % of the market share, respectively^(11)^. The OFD usage has grown rapidly, with new research showing that on average over seven million Australians aged 14 years and over are using this service in a 3-month period, up from 3·6 million in early 2020^(12)^. Within Australia, Sydney has the highest proportion of users (41 %)^(12)^. In 2022, two-fifths of people living in Australian capital cities were using this service and the primary users were millennials (born between 1981 and 1996) and Gen Z (born between 1997 and 2012)^(12,13)^. A 72 % increase in usage of OFD over 5 years in Australia was found to be driven by adults with a high income who seek convenience^(14)^. This is also supported by emerging evidence from the UK, showing least deprived areas had greater access to food outlets on OFD^(15)^. The 2019 coronavirus (COVID-19) pandemic may have further driven usage, as a report published in 2021 showed Australian consumers were spending three times more on OFD compared to pre-pandemic periods^(16)^. With easier access and more choice, there is a potential risk of increased consumption of takeaway meals that are high in saturated fat, salt, added sugars and have excessively large portion sizes^(7)^. Studies have shown that a high proportion of meals offered on OFD are poor in nutritional quality. A cross-sectional observational study in Australia and New Zealand found 86 % of popular menu items offered on the market-leading OFD platform were discretionary^(17)^. A similar study found twelve outlets available on four of the leading OFD platforms in Canada had low diet quality scores (HEI-2015) ranging from 19·95 to 50·78 out of a maximum score of 100 and did not meet healthy eating recommendations^(18)^.

Given the large proportion of unhealthy menu items offered on OFD, it is critical that consumers are provided with nutritional information such as kilojoule labelling. In New South Wales (NSW) – the most populous state in Australia with over 8·1 million people, a Menu Labelling Scheme was introduced in 2011, to raise awareness and assist consumers to make healthier choices. It mandates food outlets in NSW with more than twenty locations in NSW or more than fifty locations nationally, to display nutrition information on menus at point-of-sale, including the average energy content in kilojoules (kJs) of food items for sale and the reference statement *‘the average adult daily energy intake is 8700 kJ’* ^(19)^. An initial evaluation of the scheme targeted at 18–24-year-olds found menu labelling enhanced consumers’ understanding of average daily energy intake and led to a 519 kJ reduction in energy purchased^(19)^. A study in Australia found that compared to those without nutrition information, consumers who were provided with information selected meals with a significantly lower energy content^(20)^. In addition, a systematic review and meta-analysis further supported this, finding menu labelling to be effective in a real-world research setting, with a 420 kJ reduction in energy consumed and 325 kJ reduction in energy ordered^(21)^.

The increased demand and usage of food delivery services have led to a growing number of food outlets partnering with OFD^(17)^. However, it is unknown how well menu labelling is implemented by these online platforms. To our knowledge, there are no public health policies or nutritional labelling requirements that specifically apply to OFD platforms in Australia^(22)^. It is of further concern that food outlets with multiple locations that have fewer than twenty locations in NSW or fifty locations nationally are also likely serving energy-dense and nutrient-poor discretionary foods, and yet are exempt from the NSW Menu Labelling Scheme.

Thus, the primary aim of this study was to examine the prevalence of kJ labelling by large food outlets onto their company and third-party OFD apps in Sydney, Australia. A secondary aim was to evaluate the nutritional quality of menu items from mid-sized food outlets that do not meet the criteria for the NSW Menu Labelling Scheme.

## Methods

### Study design

This cross-sectional observational study was conducted in Sydney, Australia to assess the prevalence of kJ labelling and the nutritional quality of menu items from food outlets on OFD.

### Sample selection

#### Selection of geographical location

As millennials (born between 1981 and 1996) and Gen Z (born between 1997 and 2012) are the primary users of OFD in Sydney^(12,13)^, the top ten Local Government Areas (LGA) with the highest population of 15–44-year-olds were identified. This was achieved through a search of publicly available population-level Australian Bureau of Statistics census data^(23)^. Research has shown there is an uneven distribution of food outlets across the various suburbs by their Socio-Economic Indexes for Areas (SEIFA). SEIFA ranks areas in Australia based on their relative socio-economic advantage and disadvantage^(24)^, with unhealthiest food outlets concentrating in most disadvantaged suburbs^(17)^. Suburbs in an Australian context refer to smaller residential communities outside but close to large cities and can also include inner city and central business district areas^(25)^.

As such, from the ten LGA identified for the study, suburbs were grouped into SEIFA Index of Relative Socioeconomic Disadvantage (IRSD) to obtain an area-based measure of socio-economic status^(26)^. IRSD summarises information about the economic and social conditions of suburbs including income, educational attainment, unemployment and occupation of residents. The lower an area’s IRSD score, the greater disadvantaged residents in that area are compared with residents in other areas^(26)^. Using Microsoft Excel (version 16.66) random number generator, suburbs were randomly selected within each SEIFA decile to ensure a representative spread across Sydney. The suburbs in order of SEIFA deciles, with most disadvantaged (decile 1) to least disadvantaged (decile 10), were Fairfield, Granville, Burwood, Woodpark, Kogarah, Chifley, Dulwich Hill, Ryde, Cremorne Point and Bondi, respectively. These suburbs were used as the location for delivery to identify large and mid-sized food outlets within the third-party OFD.

#### Identification of online food delivery and food outlets

OFD assessed in this study included company and third-party ordering apps. The third-party OFD apps selected in this study were Uber Eats, Menulog and Deliveroo, as these were the most used apps by consumers in Australia^(11,12)^. In this study, the definitions of ‘large’ and ‘mid-sized’ food outlets were guided by the criteria for ‘standard food outlets’ defined by the Food Act 2003 and Food Regulation 2015^(27)^. Large food outlets were defined as those outlets with twenty or more locations in NSW or fifty or more locations in Australia, which currently are required to comply with the scheme. Mid-sized outlets were defined as those with five to nineteen locations within NSW and are yet to meet the criteria for the scheme. All food outlets identified on OFD were manually searched online for the number of locations across NSW and Australia to classify them as ‘large’ or ‘mid-sized’. This was cross-checked against an existing list of large food outlets from a NSW Food Authority evaluation report^(19)^. Company apps of these large food outlets were subsequently identified – for example, McDonald’s as a large food outlet has a separate ‘MyMacca’s’ app and Domino’s has a separate ‘Domino’s’ app.

### Data extraction

A standardised protocol was used to collect data from large and mid-sized food outlets on third-party OFD apps. Company apps of large food outlets were only assessed for kJ labelling. Two researchers with dietetic training (SC, AJ) conducted searches for the ten suburbs on each of the three OFD apps. Researchers were logged out of personal accounts to avoid possible user bias introduced by prior usage and to ensure only publicly accessible data were collected. Searches were conducted over a 3-week period from 24th August to 14th September 2022. The time window for the search was set between 18.00 and 21.00, to reduce variability of menu items offered at different times. For consistency across apps, only the name of the suburb was searched for delivery. If the app requested researchers to pin a location, the top search result was selected, which is the location that automatically appears when the suburb name is entered. If a food outlet appeared more than once in a suburb search, data from only one location were extracted. For example, when a search in a suburb (e.g. Granville) on Uber Eats showed multiple McDonald’s locations nearby, one location was randomly selected and assessed. Duplicate outlet locations within the same third-party app were excluded. For example, ‘Pizza Hut Fairfield’ was assessed once even though it was present in both searches for the suburbs of Fairfield and Woodpark on Deliveroo.

### Outcome measures

The primary outcome of this study was the prevalence of kJ labelling of menu items offered from large food outlets on their company and third-party OFD apps. The secondary outcome was the nutritional quality of menu items from mid-sized outlets and prevalence of kJ labelling of these outlets on third-party OFD.

#### Kilojoule labelling by large food outlets

For the whole menu of large food outlets, researchers observed the number of menu items with and without kJ labelling and whether it displayed the reference statement, *‘the average adult daily energy intake is 8700 kJ’ –* an additional mandatory component of the NSW Menu Labelling Scheme. All large outlets in each search were assessed, irrespective of store opening hours at time of data extraction, as menus were available for viewing. From the data collected, the proportion of menu labelling was calculated by dividing the number of menu items with kJ labelling by the total number of menu items on the menu for that location and that third party app. The prevalence of kJ labelling by large food outlets on company and third-party OFD was determined, and this was compared within and across OFD. The data were further analysed by food outlet locations. Descriptive statistical analysis was performed using Microsoft Excel (Version 16.66).

#### Nutritional quality of menu items from mid-sized food outlets

To get a better representation of what consumers could order during store opening hours, data were only collected from mid-sized food outlets that were taking orders at the time of extraction. The data collected included menu item names, pictures and descriptions. A sample of five menu items from each outlet was identified using the ‘picked for you’ section on Uber Eats which is in a prominent position at the top of the screen or webpage. Similarly, the first five menu items in the ‘popular menu section’ were collected on either Menulog or Deliveroo. These items were chosen as they are the most salient to consumers and may have increased likelihoods of being ordered, as the principles of nudging suggest^(28)^. Researchers also noted whether mid-sized food outlets had voluntarily included kJ labelling of menu items on the OFD.

Menu items collected from mid-sized outlets were classified into food and beverage categories published by Chan and colleagues^(29)^. These categories have been previously used to classify menu items on online delivery platforms^(30)^. The food and beverage categories in this classification system aligned with the food and beverage types in the Australian Dietary Guidelines classification of Five Food Groups (FFG) and Discretionary items^(4)^. Thus, the menu items could then be classified as either FFG or Discretionary.

FFG items were foods or food combinations from the five food groups: (i) vegetables and legumes/beans, (ii) fruit, (iii) grain (cereal foods), (iv) lean meats and poultry, fish, eggs, tofu, nuts and seeds and legumes/beans and (v) milk, yoghurt, cheese and their alternatives^(4)^. Discretionary food items are those that are higher in saturated fat and/or added sugars, added salt or alcohol, generally more energy dense and low in fibre^(4)^. Mixed meals or bundle meals with discretionary components were classified based on their major ingredient or item, in its respective discretionary category. For example, a burger with bacon and a burger bundle meal with chips were classified as discretionary cereal-based mixed meals.

When menu items lacked information for classification, it was classified as ‘not further defined’. Researchers (SC, AJ) cross-checked 20 % of the classification and differences were resolved by group consensus. The proportion of menu items in each category was calculated and the proportion of total discretionary items was determined.

## Results

### The prevalence of kilojoule labelling by large food outlets

#### Kilojoule labelling within each online food delivery app

**Fig. 1:**
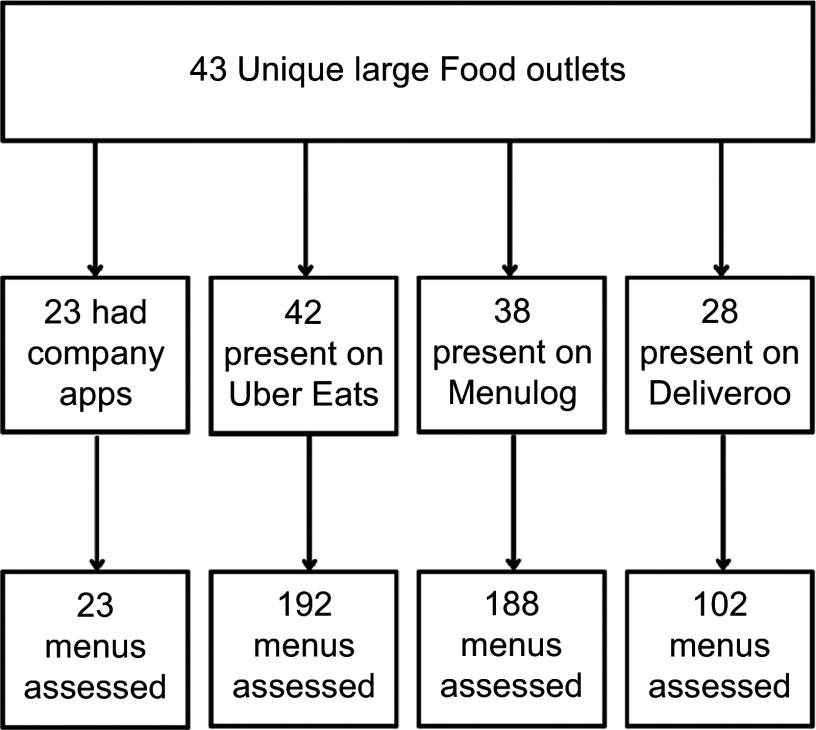
Identification of menus on online food delivery (OFD) for kJ labelling assessment from forty-three unique large food outlets in Sydney, Australia. Large food outlets were defined as those outlets with twenty or more locations in NSW or fifty or more locations in Australia that are subject to menu labelling schemes. Ten suburbs were searched on third party apps to identify large food outlets, where the menu was assessed for kJ labelling. Large food outlets varied in presence across the three third-party OFD apps and/or company apps

A total of forty-three unique large food outlets were identified from the ten suburb searches across each of the three third-party apps. Of these unique outlets, twenty-three had a company app, forty-two were present on Uber Eats, thirty-eight on Menulog and twenty-eight on Deliveroo. A total of twenty-three menus from food outlets on company apps and 482 menus from food outlets on third-party OFD were assessed as shown in Figure 1. The menu of a food outlet franchise was assessed at each different location as food outlets had more than one location within one OFD platform and across the varying OFD. For example, McDonald’s menu was assessed six times on Uber Eats due to the six different locations found. Overall, 192 menus from Uber Eats, 188 menus from Menulog and 102 menus from Deliveroo were assessed.

The median percentage of menus with kJ labelling across large food outlets on Uber Eats was 63 % (IQR: 89). Median percentage of menus with kJ labelling on Menulog was 61 % (IQR: 83). On Deliveroo, the median percentage of menus with kJ labelling was 22·5 % (IQR: 80). On company apps, the median percentage was much higher at 88 % (IQR: 27). Online supplementary material, Supplemental Table S1 provides a detailed overview of the proportion of kJ labelling across company apps and different outlet locations by each large food outlet assessed.

On company OFD apps, 35 % (8/23) of outlets had complete kJ labelling. In comparison, Uber Eats, Menulog and Deliveroo had 4·8 % (2/42), 5·3 % (2/38) and 3·6 % (1/28) of large outlets with complete kJ labelling at all locations, respectively. Online supplementary material, Supplemental Table S1 also shows that the proportion of menu items kJ labelled was inconsistent across different outlet locations within the same app, as the amount of menu items kJ labelled varied from 0 to 100 %. It was inconsistent for 55 % (23/42), 47 % (18/38) and 39 % (11/28) of large outlets on Uber Eats, Menulog and Deliveroo, respectively.

#### Kilojoule labelling across all online food delivery apps

Only one out of the 43 (2·3 %) large food outlets had complete kJ labelling across all locations and OFD apps on which it was present. In contrast, kJ labelling was completely absent on 23 % (10/43) of large outlets across all OFD apps. Of large outlets, another 23 % (10/43) had inconsistent kJ labelling across OFD, as they had menu items kJ labelled on some OFD apps but not on others.

#### Kilojoule labelling at different outlet locations

**Fig. 2:**
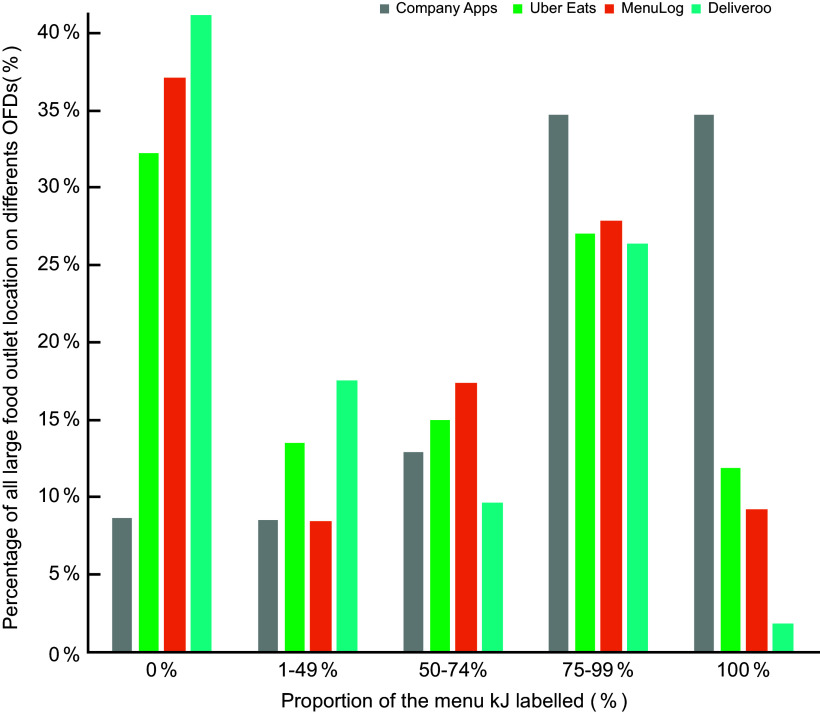
The proportion of kilojoule labelling for all large food outlet locations on different online food delivery (OFD) apps. A total of twenty-three large food outlets had a company OFD app. The number of outlet locations menus assessed on Uber Eats, Menulog and Deliveroo was 192, 188 and 102, respectively. Food outlets were categorised based on the proportion of kJ labelling, into quartiles, with the exception of ‘1–49 %’ as there was only a small percentage of outlets in this category. The proportion of menu labelling was calculated by dividing the number of menu items with kJ labelling by the total number of menu items on the menu for that location and that third party app

As large food outlets were found to have inconsistent kJ labelling within and across OFD apps, the data were further analysed by outlet locations. Figure 2 compares the proportion of kJ labelling by all large food outlet locations across their company and the three OFD apps. This figure shows that compared to company apps, where 35·0 % (8/23) of all large outlets had complete kJ labelling, only 12·0 % (23/192), 9·0 % (17/188) and 2·0 % (2/102) of outlet locations on Uber Eats, Menulog and Deliveroo, respectively, had complete kJ labelling. Two out of twenty-three outlet locations (8·7 %) did not have any form of kJ labelling on their company apps. In contrast, many more outlet locations on Uber Eats, Menulog and Deliveroo lacked any form of kJ labelling on their menu items. In respective order, kJ labelling was completely absent for 32·3 % (62/192), 37·2 % (70/188) and 41·2 % (42/102) of outlet locations on these OFD.

#### Large food outlets displaying the reference statement

Of the large food outlets that had a company app, 57·0 % (13/23) displayed the reference statement *‘The average adult daily energy intake is 8700 kJ’.* On Uber Eats, Menulog and Deliveroo, the percentage of food outlets that displayed this reference statement at all locations was 0 %, 7·9 % (3/38) and 11 % (3/28), respectively.

#### Additional observations from large food outlets

On company ordering apps, one of the outlets that had incomplete kJ labelling provided a link to access allergen information. Once researchers accessed this link, additional nutritional information could be found. Similarly, two additional food outlets on Uber Eats had a direct link to access further nutritional information. Most food outlets with customisable menu items did not include kJ labelling for those items. However, it was found that one beverage outlet had displayed kJs for each customisable add-on, for example, adding confectionery or sweets into a drink. Furthermore, one food outlet on both Uber Eats and Menulog labelled all drinks and sauces flavours with the same kJ amount, for example, ‘Coca-cola’ and ‘Coca-cola No Sugar’ were both labelled with 730 kJ even though ‘Coca-cola No Sugar’ contains < 2 kJ/100 ml^(31)^. Additionally, one food outlet had kJ labelling for all menu items, except for bottled water.

### Nutritional quality of menu items from mid-sized food outlets

A total of ninety-two mid-sized food outlets were identified across the three OFD apps. Five menu items from each food outlet were assessed for nutritional quality. Menu items in the ‘picked for you’ section were extracted from ninety outlets on Uber Eats and menu items from the ‘popular’ section were extracted from two outlets on Menulog. Eight menu items that were classified as ‘not further defined’ due to insufficient description were excluded, resulting in a total of 452 menu items. The proportion of menu items in each category is shown in Table 1.

**Table 1:** The proportion of mid-sized food outlets with menu items (*n* 452) in each category

Type of category	Food categories	*n*	%
Discretionary	Cereal-based mixed meal	104	23·0
	Meat or alternative-based mixed meal	90	19·9
	Milk-based beverages	40	8·8
	Baked good/dessert (homemade or similar)	32	7·1
	Fried potato (or similar)	26	5·8
	Iced confectionary and dairy-based desserts	26	5·8
	Vegetable-based mixed meal	17	3·8
	Sugar-sweetened beverages	10	2·2
	Total	345	76·3
Five food groups	Cereal-based mixed meal	70	15·5
	Vegetable-based mixed meal	11	2·4
	Meat or alternative-based mixed meal	9	2·0
	Meat and alternatives	8	1·8
	Beverages (juice, tea, milk/ milk alternative-based beverages)	5	1·1
	Other food (vegetables, soup, dairy and alternatives)	4	0·9
	Total	107	23·7

*Food and beverage categories are from a study published by Chan and colleagues^(29)^ (*n* 20), two categories not shown in table: cereal-based mixed meal (not further defined) and meat or alternative-based mixed meal (not further defined) as they could not be categorised into Five Food Group or Discretionary according to the Australian Dietary Guidelines^(4)^.

†Mixed meals or bundle meals with discretionary components were classified based on their major ingredient or item, in its respective discretionary category^(4, 29)^For example, a burger with bacon and a burger bundle meal with chips were classified as discretionary cereal-based mixed meals.

Discretionary items accounted for 76·3 % (345/452) of menu items. The majority of menu items were discretionary cereal-based mixed meals, which included pizzas, burgers, pasta, noodles or rice meals with discretionary components (e.g. fried chicken). The second largest discretionary item category was discretionary meat or alternative-based mixed meals. These included dishes such as fried chicken or meals with discretionary components such as meat curries made with cream.

Five food group items accounted for a total of 23·7 % (107/452) of menu items, with cereal-based mixed meals being the largest FFG category accounting for 15·5 % (70/452). These included dishes where the main component was pasta, noodles or rice without discretionary components.

#### Kilojoule labelling by mid-sized food outlets

A total of ninety-two mid-sized food outlets were identified across the three OFD apps. Mid-sized food outlets were analysed for whether they had displayed nutritional information on OFD. It was found that only two outlets – one each on Uber Eats and Menulog – had kJ labelling with 63 % and 55 % of their menu labelled, respectively.

## Discussion

This study examined the prevalence of kJ labelling on OFD menus of large and mid-sized food outlets in NSW. This study found that on all three third-party OFD apps, less than 6 % of food outlets had complete kJ labelling on their menus. Additionally, the median percentage of menus with kJ labelling was higher on company apps (88 %) compared to third party OFD platforms. Our investigations also found over 75 % of ‘picked for you’ menu items from mid-sized outlets were in the discretionary category. Taken together, the findings of this study highlight that there is considerable room for improvement of kJ labelling on OFD to help consumers make informed choices.

A main finding of this study showed kJ labelling was incomplete or absent from most food outlets on OFD. While the NSW Menu Labelling Scheme requires large food outlets to provide kJ labelling at point-of-sale^(19)^, it is evident that this information has been poorly provided on online food retail platforms. A recent study conducted in the USA similarly found more than half of restaurants offering children’s meals via online platforms were not compliant with California’s Healthy-By-Default Beverage Law (SB1192)^(32)^. This law requires restaurants that serve a children’s meal with a beverage, to make the default beverage offered to be either water, unflavoured milk or non-dairy alternatives such as soy or almond milk. Likewise, another study from the USA found only 37 % of products sold across national online grocery retailers had provided nutrition and allergen information that is historically required on food product labels^(33)^. A recent randomised controlled study found calorie labelling resulted in fewer calories being purchased on OFD^(34)^. Furthermore, between 71 and 76 % of people involved in this study supported the idea of energy labelling^(34)^. As use of these online forms of food retail continues to increase in popularity, it is critical for these food policies to be upheld and maintained in digital settings.

Menus that are only partially labelled limit a consumer’s ability to make informed choices. This study revealed inconsistencies in kJ labelling between different locations for the same franchise store and between the type of delivery service (whether through third-party couriers or company-owned). A study conducted in Canada also showed that energy labelling differed across various OFD services, with Uber Eats more likely to have provided calorie information than other platforms^(35)^. A recent Uber Eats article states that while Uber Eats are initially responsible for entering menus, once created, restaurants themselves can edit or add new menus on the app^(36)^. The ultimate responsibility of ensuring menu items have kJ labelling is therefore likely to be left to the food outlets. It is notable that Uber Eats currently does not allow menu edits to span multiple restaurant locations^(37)^, hence menus for different locations need to be created and updated individually. This may therefore explain the inconsistent labelling between different outlet locations shown in our study. Our findings suggest that delineating the responsibilities that a meal delivery application or OFD service has compared to the food outlet offering the food and drink items may be helpful in the revision or creation of new menu labelling policies.

In this study, discretionary items accounted for over three-quarters of suggested menu items from mid-sized outlets. These findings align with a study conducted by Wang & Korai where 81 % of complete menus on independent takeaway outlets were discretionary foods, and these foods were likely to be offered as value bundles^(30)^. Although the Menu Labelling Scheme is currently only applied to standard food outlets with over twenty locations in the state or over fifty locations nationally^(19)^, it may be beneficial to include these smaller ‘mid-sized’ franchise stores that do not currently meet the Scheme’s definition. Research from the USA measured the energy content of popular independent and small-chain restaurants that were not mandated to display energy values^(37)^. Investigators noticed meals from these smaller-chain restaurants averaged 49 % higher in energy than meals from the largest national chain restaurants^(37)^. In addition, an Australian study showed that the majority of products from large food outlets were classified as unhealthy based on the study’s criteria for total fat, saturated fat, sugar and Na^(38)^. Therefore, mid-sized outlets may be just as popular and nutritionally poor as larger outlets. Lowering the threshold to include mid-sized food outlets in the Scheme is a commonly suggested approach to extend the reach of menu labelling^(40–42)^. In the Australian Capital Territory, Australia, the threshold is lowered to include food outlets with seven or more locations within the territory^(43)^. Under a menu labelling scheme in the USA, this threshold includes franchises with at least twenty locations across the whole country^(44)^. It is evident that there is valid reasoning for the criteria of menu labelling schemes to be widened to include food outlets and franchises beyond an arbitrary number of locations.

### Strengths and limitations

A key strength of this study is that kJ labelling was explored across multiple market leading OFD platforms as well as company OFD. However, as of 16th November 2022, Deliveroo had announced it would no longer operate in Australia^(45)^. Previous studies that investigated OFD were limited to only one platform^(17,30)^. Moreover, the results from this study could potentially be used to inform future policy guidelines on monitoring and implementing menu labelling for online platforms.

Despite this, limitations of this study must be acknowledged. Only ten suburbs in Sydney were included in the analysis and as such, a wider reach would be needed to capture all food outlets in NSW. This study additionally included customisable items in the percentage of total kJ labelling of the menu; however, as this is not a ‘standard menu item’, the percentage of mandatory labelling could be higher for some large food outlets. Furthermore, this study only looked at the nutritional quality of five menu items from each mid-sized food outlet; hence, the results cannot be generalised for the whole food outlet, although the positioning of menu items in prominent areas such can influence their selection^(46)^.

As our study has shown there is a need for standardised approaches to menu labelling for menu boards and online platforms, other recommendations such as using the Health Star Rating or traffic light systems could similarly be adopted to provide more insight for consumers^(47,48)^. Investigation of other food outlet types such as supermarkets and convenience stores that sell products required to be kJ labelled under the scheme and can be delivered via OFD is indicated. Similar studies could also be conducted to analyse the prevalence of menus with kJ labelling in different states of Australia and world-wide.

### Conclusion

The digital food environment is continuing to influence how we access foods, with potential adverse implications for population health. Kilojoule labelling was absent or incomplete on a high proportion of online menus, particularly on third-party OFD. The inconsistency of kJ labelling across different outlet locations on OFD demonstrates the need for clear guidance for the implementation of the NSW Menu Labelling Scheme on online food retail platforms, such that customers can make informed, and ideally healthier, choices. Mid-sized outlets that are currently exempt from menu labelling should be further considered, given the high proportion of discretionary food choices. The increased usage and accessibility to discretionary foods offered via online platforms highlight the need to update public health nutrition policies on menu labelling to include the digital food environment.

## Supporting information

Cassano et al. supplementary material Cassano et al. supplementary material

## Supplementary material

For supplementary material accompanying this paper visit https://doi.org/10.1017/S1368980024000673
