# Supplementary material for: Benchmarking online food delivery applications against menu labelling laws: a cross-sectional observational analysis
Source: Public Health Nutr. 2024 Apr 1;27(1):e101. doi: 10.1017/S1368980024000673 (PMC11036439; doi:10.1017/S1368980024000673)
Supplement: Cassano et al. supplementary material [file S1368980024000673sup001.docx]

**Supplementary Material**

**Table S1: Percentage ranges of kJ labelling across company apps and different outlet locations on OFDs.**

|  | **Percentage ranges of kJ labelling (%)** | | | |
| --- | --- | --- | --- | --- |
| **Fast-food outlets assessed** (number of different locations on third-party OFDs) | **Company** | **Uber Eats** | **Menulog** | **Deliveroo** |
| **Bakers Delight** (n=12) | NA | 33-76 | 0 | NA |
| **Baskin Robbins** (n=2) | NA | 0 | NA | 0 |
| **Boost juice** (n=13) | 100 | 89 | 8 - 98 | 0-8 |
| **Broaster Chicken** (n=9) | NA | 0 | 0 | 0 |
| **Burgers with Bite** (n=3) | NA | 36-43 | 44 | 32 |
| **Cha Time** (n=10) | 100 | 0 or 100 | 99-100 | NA |
| **Coco Fresh Tea and Juice** (n=8) | 74 | 0 | 0 | NA |
| **Cold Rock** (n=2) | NA | 0 | 0 | NA |
| **Crust Pizza** (n=14) | 88 | 0 or 80 | 0 or 79 | 21-74 |
| **Dominos** (n=11) | 100 | 74-97 | 78-86 | 80-81 |
| **Donut King** (n=6) | NA | 0 | 0 | NA |
| **Fishbowl** (n=5) | NA | 0 | NA | 0 |
| **G-Free Donuts** (n=1) | NA | NA | 0 | NA |
| **Gloria Jeans** (n=3) | NA | 0-2 | 0 | NA |
| **Gong Cha** (n=15) | NA | 0 | 0 | 0 |
| **Grill'd** (n=6) | 85 | 70-79 | 69-72 | 0 |
| **Guzman y Gomez** (n=9) | 7 | 0-9 | 0 | 0-33 |
| **Hungry Jacks** (n=5) | 100 | 100 | 100 | 100 |
| **KFC** (n=15) | 83 | 75-92 | 55-74 | 79-83 |
| **Mad Mex** (n=6) | 0 | 0 | 0 | 0 |
| **McDonalds** (n=16) | 100 | 100 | 100 | 0 |
| **Muffin Break** (n=5) | NA | 0 | 48-92 | 0 |
| **Nandos** (n=2) | 90 | 64 | 62 | 64 |
| **Oliver Brown** (n=9) | NA | 0-65 | 0-100 | 0 |
| **Oporto** (n=15) | NA | 74-97 | 97 | 96-98 |
| **Pizza Hut** (n=13) | 100 | 55-100 | 0-66 | 0-76 |
| **Rashays** (n=4) | 0 | 0 | 0 | 0 |
| **Red Rooster** (n=8) | 100 | 64-78 | 77-80 | 78 |
| **Roll'd** (n=6) | 72 | 59-68 | 49-75 | 53-60 |
| **San Churro** (n=2) | 66 | 50-52 | 74 | NA |
| **Schnitz** (n=2) | 46 | 6 | NA | NA |
| **ShareTea** (n=13) | NA | 0 | 24-84 | 71-86 |
| **Soul Origin** (n=12) | 80 | 0-66 | 67-71 | 0 |
| **Starbucks** (n=4) | NA | 23-92 | NA | NA |
| **Subway** (n=16) | 100 | 3-16 | 0-89 | 0 |
| **Sushi Hub** (n=7) | NA | 30-79 | 78-85 | 0 |
| **Sushi Train** (n=4) | 92 | 79-80 | 84-85 | 81-83 |
| **The Cheesecake shop** (n=6) | NA | 53-84 | 64-81 | NA |
| **The Coffee Club** (n=6) | NA | 65 | 67-86 | 56-57 |
| **Top Juice** (n=7) | 90 | 90-93 | 87 | NA |
| **Wing Street** (n=2) | 83 | 92-93 | NA | NA |
| **Yogurberry** (n=4) | NA | 0 | 0 | NA |
| **Zambrero** (n=2) | NA | 9 | 9-11 | NA |

NA= Not applicable as the large food outlet did not have a company food ordering app or was unavailable on a third-party OFD.
